# Gene Expression and K^+^ Uptake of Two Tomato Cultivars in Response to Sub-Optimal Temperature

**DOI:** 10.3390/plants9010065

**Published:** 2020-01-03

**Authors:** Huan Gao, Wanji Yang, Chunxia Li, Xingang Zhou, Danmei Gao, Muhammad Khashi u Rahman, Naihui Li, Fengzhi Wu

**Affiliations:** 1 Department of Horticulture, Northeast Agricultural University, Harbin 150030, China; gh18040102@163.com (H.G.); lcx198238@163.com (C.L.); xgzhou@neau.edu.cn (X.Z.); dmgao2019@neau.edu.cn (D.G.); khashiurahman@yahoo.com (M.K.uR.); linaihui1992@aliyun.com (N.L.); 2 Key Laboratory of Biology and Genetic Improvement of Horticultural Crops (Northeast Region), Ministry of Agriculture, Northeast Agricultural University, Harbin 150030, China; 3 Department of Computer and Information Engineering, Heilongjiang University of Science and Technology, Harbin150030, China; fen1212dou@163.com

**Keywords:** sub-optimal temperature, tomato, transcriptome, potassium, uptake

## Abstract

Sub-optimal temperatures can adversely affect tomato (*Solanum lycopersicum*) growth, and K^+^ plays an important role in the cold tolerance of plants. However, gene expression and K^+^ uptake in tomato in response to sub-optimal temperatures are still not very clear. To address these questions, one cold-tolerant tomato cultivar, Dongnong 722 (T722), and one cold-sensitive cultivar, Dongnong 708 (S708), were exposed to sub-optimal (15/10 °C) and normal temperatures (25/18 °C), and the differences in growth, K^+^ uptake characteristics and global gene expressions were investigated. The results showed that compared to S708, T722 exhibited lower reduction in plant growth rate, the whole plant K^+^ amount and K^+^ net uptake rate, and T722 also had higher peroxidase activity and lower K^+^ efflux rate under sub-optimal temperature conditions. RNA-seq analysis showed that a total of 1476 and 2188 differentially expressed genes (DEGs) responding to sub-optimal temperature were identified in S708 and T722 roots, respectively. Functional classification revealed that most DEGs were involved in “plant hormone signal transduction”, “phenylpropanoid biosynthesis”, “sulfur metabolism” and “cytochrome P450”. The genes that were significantly up-regulated only in T722 were involved in the “phenylpropanoid biosynthesis” and “plant hormone signal transduction” pathways. Moreover, we also found that sub-optimal temperature inhibited the expression of gene coding for K^+^ transporter SIHAK5 in both cultivars, but decreased the expression of gene coding for K^+^ channel AKT1 only in S708. Overall, our results revealed the cold response genes in tomato roots, and provided a foundation for further investigation of mechanism involved in K^+^ uptake in tomato under sub-optimal temperatures.

## 1. Introduction

Tomato (*Solanum lycopersicum*), which originated in sub-tropical areas [1], is sensitive to chilling temperature [2,3]. However, its production areas usually experience sub-optimal temperatures (which range between two thresholds, a minimum (8–12 °C) and an optimum (25–27 °C)) [4,5,6]. Sub-optimal temperatures affect the vegetative and fruit growth of tomato plants, which ultimately leads to decline in the yield [1]. Hence, to improve tomato production and breeding under these conditions, it is necessary to understand the mechanisms underlying tomato responses to sub-optimal temperatures. 

Plants can respond and tolerate to cold stress by changing several biochemical processes and traits features [7]. These changes include alterations in the composition, structure, and function of the plasma membrane; synthesis of cryoprotectant molecules, including soluble sugars and low-molecular-weight nitrogenous compounds, such as proline; and increases in the scavenging activity of reactive oxygen species (ROS) [8,9]. Peroxidase (POD) is one of the most important antioxidative enzymes, and can alleviate oxidative damage by scavenging ROS under stress conditions [10,11]. Cold temperatures also induce cascades of alterations in metabolic pathways, including the phenylpropanoid pathway, and in the activities of antioxidant enzymes [12]. As one of the most important inorganic osmotica in plants, potassium (K) plays key role in establishing the osmotic adjustment ability [13,14]. The available evidence suggest that K improves plant survival rate under cold stress by reducing ROS accumulation and increasing antioxidant levels [15,16]. A significant negative correlation has been found between the leaf K concentration and frost damage, and an adequate K supply can effectively increase the frost resistance of plants, whereas K deficiency can increase the susceptibility of plants to low temperatures [17,18,19]. The K requirement of greenhouse tomato plants is very high for vegetative growth and fruit production [20], but little is known regarding the K^+^ uptake characteristics of two tomato cultivars differing in cold resistance under sub-optimal temperature conditions. 

In plant roots, K^+^ uptake from the outer environment is mainly mediated by K^+^ channels and transporters [21,22,23]. All abiotic stress results in disturbance to K^+^ homeostasis and provokes a feedback control on K^+^ channels and transporters expression and post-translational regulation of their activity, optimizing K^+^ absorption and usage [24,25,26]. However, few studies have focused on the expression of genes related to K^+^ uptake and translocation during exposure to sub-optimal temperature stress. Although some studies have reported that low temperature decreases the uptake [27] and influx [26] of K^+^, it is still unclear whether sub-optimal temperatures affect K^+^ uptake and translocation by affecting the expression of genes coding for K^+^ channels and transporters. 

RNA sequencing (RNA-seq) is a critical method for profiling global gene expression in response to biotic and abiotic stress [28,29]. In tomato, transcriptome profiling has been used for comparative analysis of expression of defense related genes between two tomato cultivars with different resistances to salt, cold and disease stress [30,31,32]. However, to the best of our knowledge, a transcriptome analysis of tomato under sub-optimal temperature conditions that focuses on the root system has not been reported yet.

In this study, we profiled differences in plant growth, K^+^ uptake, and gene expression changes by making comparison between one cold-sensitive and one cold-tolerant tomato cultivar exposed to sub-optimal temperature conditions. We hypothesized that (1) under sub-optimal temperature conditions, the plant growth rate (PGR) and the POD activity of roots in cold-tolerant cultivar would be higher than that in the cold-sensitive cultivar; and (2) the cold-tolerant cultivar would exhibit enhanced cold tolerance by regulating not only the expression of genes related to cold tolerance in roots, but also K^+^ uptake. As mentioned earlier, K^+^ uptake from outside is mainly mediated by K^+^ channels and transporters; thus, we also hypothesized that (3) sub-optimal temperatures could inhibit the expression of genes coding for K^+^ channels and transporters in roots, and that the inhibitory effect on cold-sensitive tomato would be stronger than that on cold-tolerant tomato.

## 2. Results

### 2.1. Plant Dry Weight and Root Morphology

**Figure 1:**
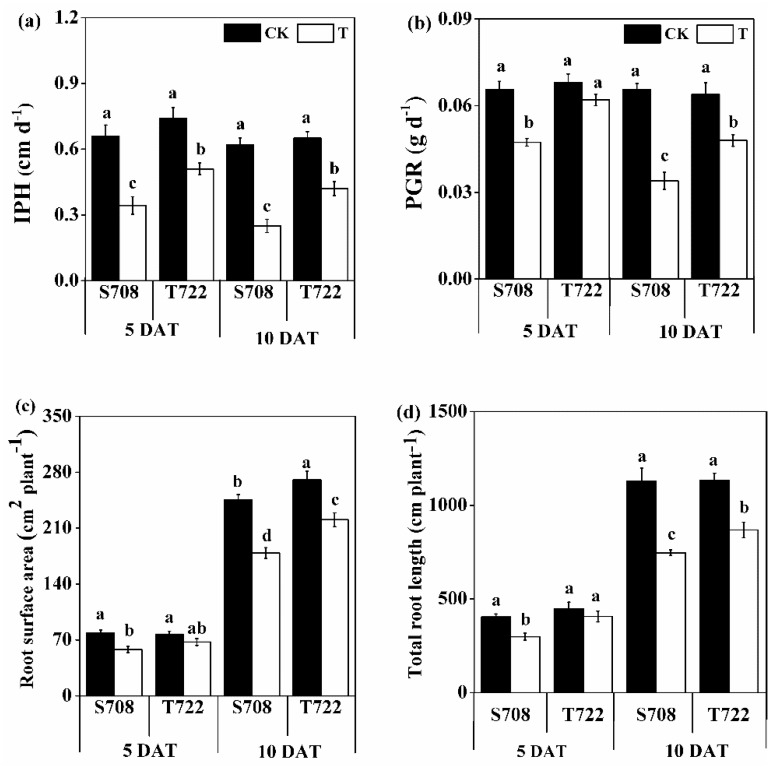
Phenotypic response of the two tomato cultivars to sub-optimal temperature. (**a**) Increase in plant height (IPH), (**b**) plant growth rate (PGR), (**c**) root surface area, and (**d**) total root length of S708 and T722 cultivars grown at normal temperature (CK, 25/18 °C) and sub-optimal temperature (T, 15/10 °C) at 5 and 10 DAT. Data are the means with standard deviation shown by vertical bars (n = 3 representing 3 biological replicates). The different small letters above the bars represent significant differences attested by the Tukey’s HSD test (*p* < 0.05).

After 5 and 10 days after treatment (DAT), the increase in plant height (IPH), plant growth rate (PGR), root surface area and total root length of the two tomato cultivars under sub-optimal temperature were measured. Temperature significantly affected IPH, PGR, root surface area and total root length (Table 1). Compared with the normal temperature treatment (CK), sub-optimal temperature (T) significantly decreased the IPH, PGR, root surface area and total root length in S708 at 5 DAT, and decreased these parameters in both tomato cultivars at 10 DAT. Under sub-optimal temperature conditions, the magnitude of decreases of IPH and PGR in S708 was larger than that in T722 at 5 and 10 DAT (Figure 1). There was a significant two-way interaction between temperature and cultivar for IPH and PGR at 10 DAT (Table 1).

### 2.2. MDA Content and POD Activity 

**Figure 2:**
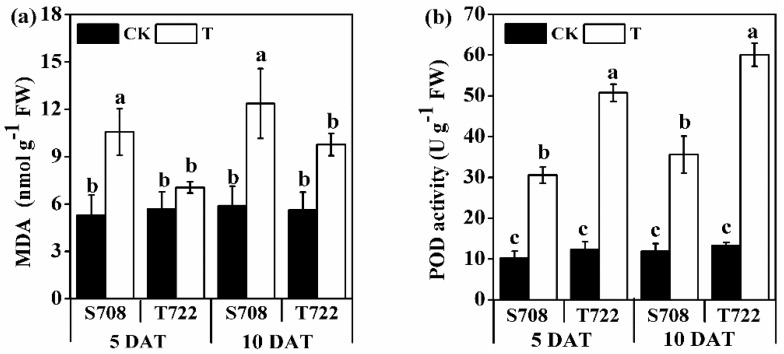
Physiological responses of the two tomato cultivars to sub-optimal temperature. (**a**) MDA content and (**b**) POD activity of S708 and T722 cultivars grown at normal temperature (CK, 25/18 °C) and sub-optimal temperature (T, 15/10 °C) at 5 and 10 DAT. Data are the means with standard deviation shown by vertical bars (n = 3 representing 3 biological replicates). The different small letters above the bars represent significant differences attested by the Tukey’s HSD test (*p* < 0.05).

Temperature significantly affected malondialdehyde (MDA) content and POD activity at 5 and 10 DAT (Table 1). Both cultivars exhibited an increase in MDA content after treated with sub-optimal temperature (T) at 10 DAT (Figure 2), and the magnitude of increase in MDA content in S708 was larger than that in T722 at 5 and 10 DAT. Sub-optimal temperature significantly increased the POD activity in both cultivars at both sampling dates, and the magnitude of increase in POD activity in T722 was larger than that in S708. There was a significant two-way interaction between temperature and cultivar for POD activity at 5 and 10 DAT (Table 1).

### 2.3. K^+^ Content in Different Organs and K^+^ Uptake by Whole Plants

Sub-optimal temperature treatment (T) significantly increased the roots K^+^ content in both tomato cultivars at 5 DAT, although the roots K^+^ content did not differ between the normal (CK) and sub-optimal temperature treatments in both cultivars at 10 DAT (Table 2). Temperature significantly affected K^+^ content in stem. The stem K^+^ contents obtained for S708 at 5 and 10 DAT at sub-optimal temperature were 8.73% and 13.45% lower, respectively, than those obtained with the normal temperature treatment, whereas the rates obtained for T722 were 5.99% and 5.03% lower, respectively, than those obtained with the normal temperature treatment. Sub-optimal temperature significantly reduced the leaves’ K^+^ content in S708 at 10 DAT, although the leaf K^+^ content did not differ between the normal and sub-optimal temperature treatments in both cultivars at 5 DAT. For each cultivar, the whole plant K^+^ amount was significantly lower under sub-optimal temperature treatment than under normal temperature treatment at both sampling dates. The whole plant K^+^ amounts obtained for S708 at 5 and 10 DAT with sub-optimal temperature were 45.01% and 36.98% lower, respectively, than those obtained with the normal temperature treatment, whereas the rates obtained for T722 at 5 and 10 DAT with sub-optimal temperature were 17.26% and 19.09% lower, respectively, than those obtained with the normal temperature treatment. Moreover, at the same sampling date, the whole plant K^+^ amount obtained for T722 was higher than that obtained for S708 under normal and sub-optimal temperature conditions (Table 2).

### 2.4. K^+^ Net Uptake Rate and K^+^ Transportation Ratio

**Figure 3:**
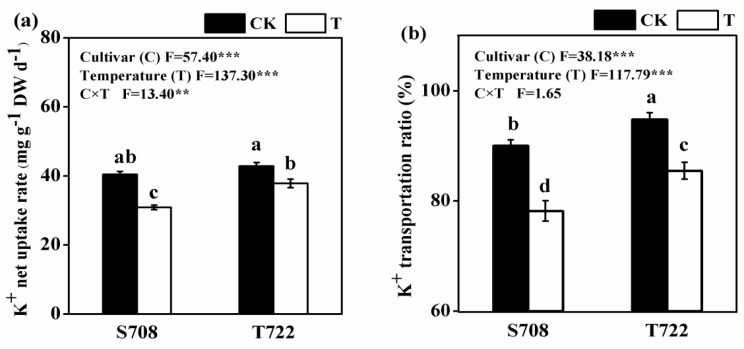
K^+^ net uptake rate and K^+^ transportation ratio of S708 and T722 cultivars under normal (CK, 25/18 °C) and sub-optimal temperature (T, 15/10 °C) conditions. (**a**) K^+^ net uptake rate during days 5–10 of growth. (**b**) The percentage of net increased K^+^ in shoots to net increased total K^+^ in the entire plant during the days 5–10 of growth. Data are the means with standard deviation shown by vertical bars (n = 3 representing 3 biological replicates). The different small letters above the bars represent significant differences attested by the Tukey’s HSD test (*p* < 0.05). *F*-values from two-way ANOVA analysis of effects of cultivar and temperature treatment on K^+^ net uptake rate and K^+^ transportation ratio. *p ≤ 0.05, **p ≤ 0.01, ***p ≤ 0.001.

Sub-optimal temperature significantly decreased the K^+^ net uptake rate and K^+^ transportation ratio in both tomato cultivars during 5–10 days (Figure 3). Compared with normal temperature treatment, the magnitude of decreases in K^+^ net uptake rate and K^+^ transportation ratio under sub-optimal temperature in S708 was larger than that in T722. There was a significant two-way interaction between temperature and cultivar for K^+^ net uptake rate (Figure 3). 

### 2.5. K^+^ Flux Rate 

**Figure 4:**
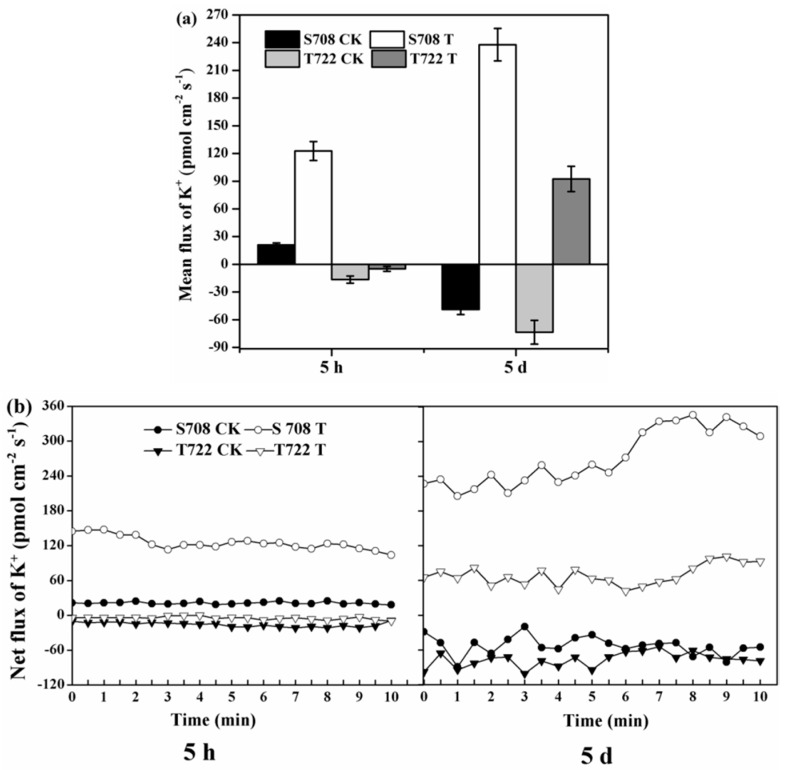
Net flux of K^+^ in root tips of S708 and T722 cultivars under normal (CK, 25/18 °C) and sub-optimal temperature (T, 15/10 °C) conditions. After being treated with normal and sub-optimal temperatures for 5 hours and 5 days, a continuous flux recording of 10 min was conducted. (**a**) Mean fluxes of K^+^ and (**b**) net flux of K^+^ in both cultivars within the measuring periods. The data were collected every 6 s during the 10 min measuring period. The positive values in the figures represent K^+^ efflux and negative values represent K^+^ influx.

At 5 hours after treatment, the mean flux of K^+^ towards the external medium in T722 was negative under normal temperature (CK) and sub-optimal temperature (T), indicating the occurrence of K^+^ influx in the roots (Figure 4a). In contrast, the mean flux of K^+^ obtained in S708 was positive under normal temperature and sub-optimal temperature conditions, indicating the occurrence of K^+^ efflux in the roots. At 5 DAT, K^+^ influx occurred in both cultivars under normal temperature condition; however, the K^+^ efflux of both cultivars increased markedly under sub-optimal temperature conditions, and a more pronounced increase was observed in S708 (Figure 4a). At 5 hours after treatment, the net flux of K^+^ in both cultivars exhibited a stable and constant flux during the 10 min measurement period under normal and sub-optimal temperature conditions (Figure 4b). At 5 DAT, the trend of net flux of K^+^ in S708 was similar to that in T722 under normal and sub-optimal temperature conditions. The K^+^ efflux increased from 227 to 308 pmol cm^−2^ s^−1^ in the S708 cultivar, and from 65.57 to 92.62 pmol cm^−2^ s^−1^ in T722 during the 10 min measurement period under sub-optimal temperature condition (Figure 4b).

### 2.6. Summary of RNA-Seq Data

To investigate the alterations in root gene expression regulated by sub-optimal temperatures, an RNA-Seq analysis of roots taken from S708 and T722 plants at 5 DAT was conducted using Illumina sequencing technology. More than 40 million (M) single-end reads were generated for each library, and the total number of clean reads ranged from 40 to 60 M, with a mapping rate over 95% (Table S1).

#### 2.6.1. DEGs in Response to Sub-Optimal Temperature

**Figure 5:**
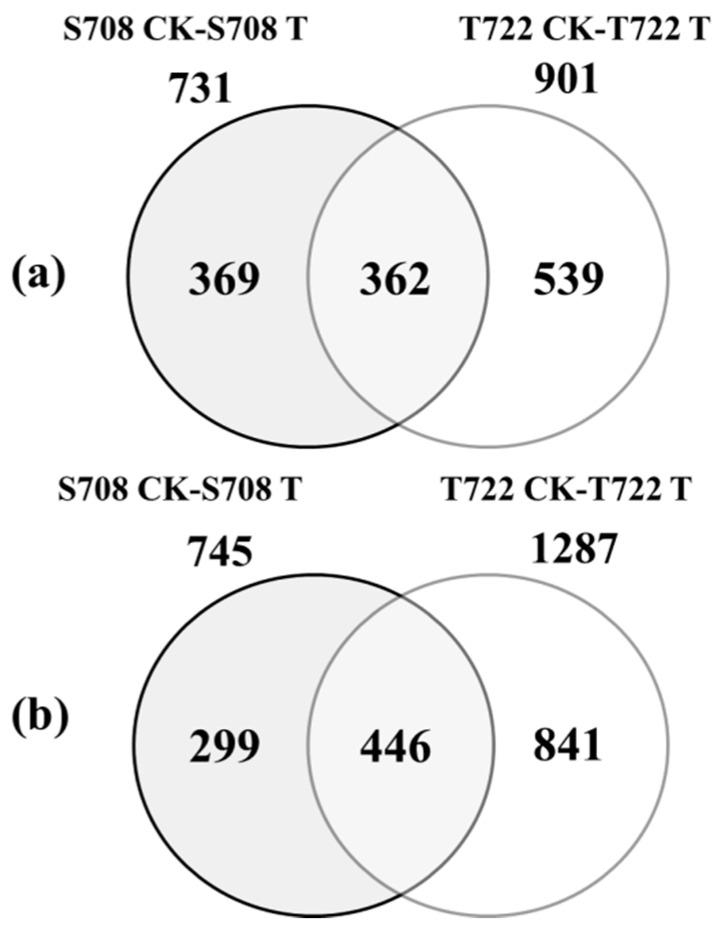
Venn diagrams showing differentially up-regulated (**a**) or down-regulated (**b**) genes in S708 CK vs. S708 T and T722 CK vs. T722 T. S708 CK and T722 CK: S708 and T722 tomato seedlings grown under normal temperature condition (CK, 25/18 °C), S708 T and T722 T: S708 and T722 tomato seedlings grown under sub-optimal temperature conditions (T, 15/10 °C). The numbers above the circles indicate the total number of up- or down-regulated genes at sub-optimal temperature in each cultivar.

The significant DEGs were identified by the criteria of at least a 2-fold change, and the *p*-value of the false discovery (FDR) correction had to be less than 0.05 between the treated and control groups of S708 and T722 cultivars. The FPKM and FDR values and the fold-changes for each gene of the S708 and T722 cultivar are shown in Supplementary Tables S2 and S3, respectively. The results showed that the number of up-regulated or down-regulated DEGs was much higher in T722 than in S708 (Figure 5). Moreover, 731 and 901 genes were up-regulated in S708 and T722, respectively, and 362 genes were common in both tomato cultivars (Figure 5a); 745 and 1287 genes were down-regulated in S708 and T722, respectively, and 446 genes were common in both tomato cultivars (Figure 5b). 

#### 2.6.2. GO and KEGG Enrichment Analysis of DEGs

To unravel the significantly altered biological processes upon cold stress, the DEGs of the two tomato cultivars were subjected to the GO term enrichment analysis [33]. Some stress-related GO biological process terms, such as ‘oxidation-reduction process’, ‘hydrogen peroxide metabolic process’ and ‘hydrogen peroxide catabolic process’ were significantly enriched (*p* < 0.001) in both tomato cultivars (Table S4). More GO terms were significantly enriched (*p* < 0.001) in T722. Among these, two were involved in stress responses, including ‘response to stimulus’ and ‘phenylpropanoid metabolic process’ and four were involved in hormone responses, including ‘hormone metabolic process’, ‘hormone biosynthetic process’, ‘cytokinin biosynthetic process’ and ‘cytokinin biosynthetic process’ (Table S4).

To study the biological pathways associated with the DEGs, a KEGG pathway analysis of all the DEGs was performed. We summarized the top 10 enriched KEGG pathways, and the up- and down-regulated DEGs in the KEGG pathway are shown in Table S5. In the main text, we describe four significantly enriched KEGG pathways. The DEGs in the S708 CK-S708 T and T722 CK-T722 T groups were significantly enriched in four metabolic pathways with *p*-values < 0.05: “plant hormone signal transduction” (24 DEGs/38 DEGs), “phenylpropanoid biosynthesis” (28 DEGs/42 DEGs), “sulfur metabolism” (8 DEGs/10 DEGs) and “cytochrome P450” (13 DEGs/16 DEGs) (Table 3). 

Among the DEGs, 11 genes in the significantly enriched KEGG pathway “plant hormone signal transduction” were significantly induced by sub-optimal temperature in T722 (Table 4), and 12 peroxidase-related genes in the significantly enriched KEGG pathway “phenylpropanoid biosynthesis” were significantly induced by sub-optimal temperature in T722 (Table 5).

#### 2.6.3. Expression Analysis of Genes Related to K^+^ Uptake and Translocation 

Solyc03g083320.2 encoding a homologue of *Oryza sativa* calcineurin B-like protein OsCBL7, Solyc09g042660.2 and Solyc03g006110.2 encoding two homologues of *O. sativa* CBL-interacting protein kinase OsCIPK18 and OsCIPK5 were induced by sub-optimal temperature only in T722 (Table 6). Solyc09g005220.1 encoding a homologue of *Arabidopsis* K^+^ channel AKT1 was inhibited during exposure to sub-optimal temperature, and this inhibitory effect was stronger in S708 than in T722. Moreover, Solyc12g005670.1 encoding *S. lycopersicum* K^+^ transporter SIHAK5, Solyc06g051830.1 encoding a homologue of *O*. *sativa* K^+^ transporter OsHAK26, and Solyc08g007060.2 encoding a homologue of *Arabidopsis* nitrate transporter AtNRT1.5 were repressed in both cultivars under sub-optimal temperature condition (Table 6).

#### 2.6.4. Expression Analysis of Aquaporin Genes and Cold Tolerance-Related Genes

Two genes encoding aquaporins were repressed in both cultivars under sub-optimal temperature conditions (Table S6). Through the analysis of cold resistance-related gene expression during sub-optimal temperature stress, we found that two genes encoding proline dehydrogenase were repressed in T722; of these, one proline dehydrogenase gene was repressed in S708. Moreover, three genes encoding mitogen-activated protein kinase (MAPK) were induced only in T722 under sub-optimal temperature conditions (Table S6). 

### 2.7. Verification of RNA-Seq Data

**Figure 6:**
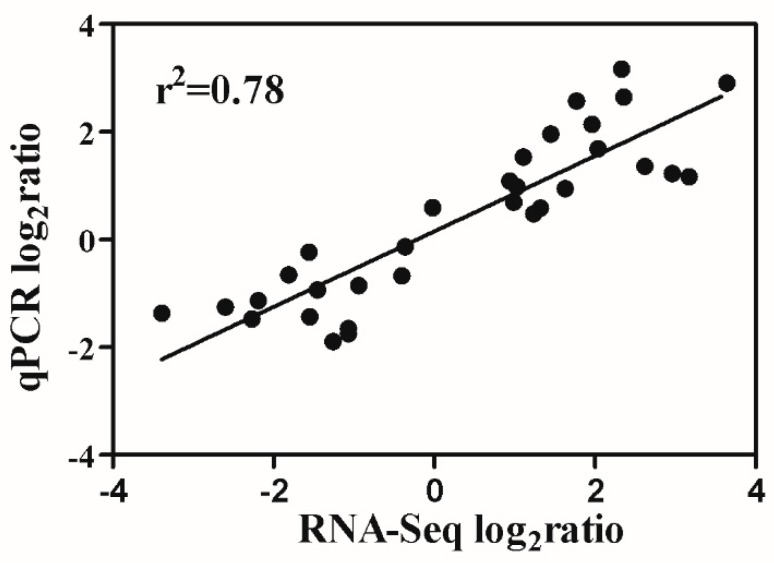
Correlation analysis of gene expression values obtained from RNA-seq and data obtained using real-time qPCR. All qPCR reactions were performed in three biological replicates. Values are the log_2_ratio(T/CK) for genes. The determined coefficient (r^2^) is indicated in the figure.

The RNA-seq data was verified through a quantitative real-time PCR analysis of sixteen genes, which were selected from the following four groups: (i) the genes related to K^+^ uptake and translocation, (ii) down-regulated aquaporin genes in both cultivars, (iii) up-regulated genes involved in the “phenylpropanoid biosynthesis” pathway in both cultivars, and (iv) up-regulated genes involved in the “plant hormone signal transduction” pathway in both cultivars under sub-optimal temperature condition. qPCR results revealed that gene expression trends were significantly similar (r^2^ = 0.78) to those from the RNA-seq data, indicating that our RNA-seq results were reliable (Figure 6). 

## 3. Discussion

Decreasing the greenhouse temperatures affects different aspects of tomato growth and development [1]. The comparison performed in this study showed that the phenotype of S708, including the IPH, and PGR was more significantly affected than that of T722 by sub-optimal temperature treatment (Figure 1). The results described above suggest that the T722 cultivar was more tolerant to sub-optimal temperature than the S708 cultivar. Moreover, the T722 cultivar had a more developed root system than the S708 cultivar under sub-optimal temperature condition (Figure 1c,d), which indicated that the T722 cultivar has a stronger nutrient uptake ability than the S708 cultivar [34]. MDA content was used to evaluate the cell membrane damage [35]. In our study, the MDA content was significantly higher in S708 than in T722 under sub-optimal temperature condition (Figure 2a). This result suggested that the T722 suffered less membrane damage than S708 under sub-optimal temperature stress.

Low temperature may lead to osmotic stress because low temperature impairs water absorption and transport [36]. K^+^ plays a key role in the establishment of a plant’s ability to adjust to osmotic changes and in improving the cold tolerance of plants [14]. However, K^+^ uptake characteristics of tomatoes in response to sub-optimal temperatures are still not very clear. Our results showed that the whole plant K^+^ amount and the net K^+^ uptake rate were significantly higher in T722 than in S708 under sub-optimal temperature condition (Table 2 and Figure 3a). Many studies have indicated that there was a significant positive correlation between the K^+^ amount and plant resistance to abiotic stress [17,18,19]. A sufficient K supply can reduce the formation of ROS in plant cells and alleviate the damage to plants exposed to chilling or freezing stress [15]. Therefore, we deduced that the higher K^+^ amount observed in T722 might contribute to the defense of the plants against sub-optimal temperature stress. Moreover, we found that sub-optimal temperature induced a reduction in the K^+^ transportation ratio in both cultivars during the 5–10 days period (Figure 3b). This reduction might have caused the K^+^ content in roots to significantly increase and that in stems to significantly decrease in both cultivars at 5 DAT (Table 2). 

To investigate the differences in gene expression between the T722 and S708 cultivars in response to sub-optimal temperature, we performed a comparative transcriptome analysis. The overall number of DEGs in cold-tolerant cultivar was higher than that in cold-sensitive cultivar at 5 DAT (Figure 5). This result is different from the previous study [35], and this might be related to the characteristics of the cultivar used in experiment. Different cultivars showed different cold responses at the transcriptional level. The DEGs may reveal the difference in the response to cold stress between the S708 and T722 cultivars. GO enrichment analysis is helpful for highlighting the main biological processes in response to stress environment [37]. In our study, we found that the genes involved in ROS homeostasis and hormone metabolic were differentially expressed between S708 and T722 under sub-optimal temperature condition. These results showed that hormone and ROS may plant key roles in regulating gene expression in response to sub-optimal temperature stress.

The cultivar-specific DEGs in T722 were considered crucial to its higher cold tolerance. Among them, most DEGs were involved in the “plant hormone signal transduction” and “phenylpropanoid biosynthesis” pathways. Plant hormones are known to regulate plant growth and development and thus allow the adaptation of plants to abiotic stress [38]. Our results showed that 11 genes in the significantly enriched KEGG pathway, “plant hormone signal transduction” were induced only in T722 at 5 DAT (Table 4). Among these genes, two auxin-related genes, including Solyc09g065850.2, which encodes indole-3-acetic acid (IAA)-induced protein IAA3, were up-regulated only in T722. In *Arabidopsis*, mutation of this gene can affect auxin-dependent root growth and lateral root formation [39]. Moreover, Solyc04g078470.2, which encodes a homologue of *Arabidopsis* CYCD3, responds to cytokinins and is required for proper secondary root thickening [40]. These results might be linked to the stronger root system in T722 than in S708 under sub-optimal temperature stress. Solyc08g008600.2, involved in the jasmonic acid (JA) signaling pathway and encodes a transcription factor MYC2, which positively regulates the formation of lateral roots [41] and tolerance to oxidative stress in *Arabidopsis* [42]. This gene was strongly induced by sub-optimal temperature only in T722, which suggested that its up-regulation might contribute to cold tolerance in tomato. This result is consistent with the findings obtained in previous studies [15], which showed that JA might act as a positive regulator in response to cold stress in tomato. Three genes (Solyc01g106640.2, Solyc10g085960.1 and Solyc09g007020.1) involved in the salicylic acid (SA) signaling pathway that encode the pathogenesis-related (PR) protein PR1 were more strongly induced by sub-optimal temperature in T722 than in S708 (Table 4). PR proteins such as β-1,3-glucanase exhibit antifreeze activity and even prevent the formation of intracellular ice [43]. These results showed that the plant hormones IAA, SA and JA might play their role in regulating root growth and in resisting sub-optimal temperature in T722.

Further results showed that 12 peroxidase related genes involved in the significantly enriched KEGG pathway “phenylpropanoid biosynthesis” were significantly induced only in T722 at 5 DAT (Table 5). This result is consistent with the results of POD activity which significantly increased in T722 under sub-optimal temperature (Figure 2b). ROS are produced by plants upon exposure to low-temperature stress [44,45], and peroxidase is one of the most important antioxidative enzymes, and can alleviate oxidative damage by scavenging ROS under stress conditions [10,11]. Moreover, we found that three genes encoding mitogen-activated protein kinase (MAPK) (Solyc12g005360.1, Solyc02g090980.1 and Solyc05g008020.2) were induced only in T722 during exposure to sub-optimal temperatures (Table S6). MAPK can enhance *Nicotiana tabacum* total peroxidase (POD) activity, thereby inducing less accumulation of H_2_O_2_ and alleviation of ROS-mediated injuries [46], which might at least partly explain the large number of peroxidase related genes that were up-regulated in T722. K^+^ efflux is an important physiological phenomenon, that is usually detected almost instantaneously after the application of a stress factor and lasts from a few minutes to several hours [47]. Our results showed that the T722 cultivar had a lower K^+^ efflux rate than S708 cultivar at 5 hours and 5 days after treatment (Figure 4), which is consistent with the previous results reported by Chen et al. (2005) [48], who indicated that the cold-tolerant tomato root system has a strong ability to maintain K^+^. K^+^ efflux is mainly induced by stresses and ROS. The present results suggested that the sub-optimal temperature treatment induced the expression of genes coding for MAPK and peroxidase in T722, and increased the POD activity, which might contribute to ROS scavenging and reduced loss of K^+^. 

It worth noting that almost the same number of peroxidase-related genes involved in the “phenylpropanoid biosynthesis” pathway were repressed in T722 (Table S5). Peroxidases are involved in lignification, cell elongation, stress defense and seed germination. The diverse peroxidase activities facilitate opposing reactions in plants, such as generation/scavenging of ROS and loosening/stiffening of the cell wall [49]. Similar results were also found in cytochrome P450 pathway; there were lots of up- and down-regulated glutathione S transferases (GSTs) involved in cytochrome P450 pathway, and these genes play roles in detoxifying oxidative-stress metabolites [50]. The expression of defense genes has negative effects on plant growth, which to some degree counterbalances their positive effects [34]. So we deduced that the up- and down-regulated defense related genes in T722 might help to maintain energy balance. Moreover, our results showed that eight sulfur metabolism-related genes were up-regulated in both cultivars. Sulfur assimilation is a platform for the biosynthesis of sulfur-containing defense compounds (SDCs), which are crucial for the survival of plants under biotic and abiotic stress [51].

To clarify the change of K^+^ content in response to sub-optimal temperatures, the expression of genes coding for K^+^ uptake and translocation was analyzed (Table 6). The results showed that Solyc12g005670.1, the K^+^ transporter SIHAK5, and a homologue of the *O. sativa* K^+^ transporter HAK26 (Solyc06g051830.1), was repressed in both cultivars at 5 DAT. Moreover, under sub-optimal temperature conditions, a homologue of the *Arabidopsis* K^+^ channel AKT1 (Solyc09g005220.1) was repressed only in S708. Similarly, the expression of another gene coding for *S. lycopersicum* K^+^ channel LKT1 (Solyc12g006850.1) was down-regulated 2.11-fold in S708 and 1.44-fold in T722 (Table 6). The Shaker type K^+^ channel LKT1 in tomato belongs to the *Arabidopsis* AKT1 subfamily, and HAK5 belongs to the KT/HAK/KUP transporter family, which plays an important role in K^+^ uptake [52,53,54]. Then, we verified the expression of these genes by qRT-PCR, and they were consistent with the RNA-seq data, despite the differential expression folds (Figure 6). 

The phosphorylation of K^+^ channels and transporters is the most important mechanism for the regulation of their function [22]. Several studies have demonstrated that the CBL-CIPK complex can regulate not only the expression of *AKT1*, but also the expression of *AtHAK5* [55,56,57,58]. Our results show that a homologue of the *O. sativa* calcineurin B-like protein CBL (Solyc03g083320.2) and two homologues of the *O. sativa* CBL-interacting protein kinase CIPK (Solyc09g042660.2 and Solyc03g006110.2) were induced only in T722 (Table 6). Our findings suggest that sub-optimal temperatures might repress K^+^ net uptake rate and K^+^ influx by inhibiting the expressions of gene coding for K^+^ channel and transporter and the CBL-CIPK pathway may be more active in T722 during sub-optimal temperature stress. Moreover, we found that Solyc08g007060.2, which encodes a homologue of the *Arabidopsis* nitrate transporter NPF7.3/NRT1.5, was inhibited in both cultivars by the sub-optimal temperature treatment (Table 6). The encoded protein, which can regulate K^+^ translocation to the shoots in *Arabidopsis* [59,60], was repressed by sub-optimal temperature. That may explain the decreased K^+^ transportation ratio in both cultivars during the 5–10 days after treatment (Figure 3b). In addition, there are close couplings between aquaporins and K-channel transporters in water uptake of roots [20] and osmoregulation [61]. Our transcriptome and qPCR analysis showed that sub-optimal temperature inhibits not only genes related to K^+^ uptake and translocation, but also aquaporins (Table S6 and Figure 6), which may be another reason for the increased K^+^ accumulation in roots under sub-optimal temperature conditions. Extensive studies have shown the roles of K^+^ uptake and transport related genes in resistance to salt and low potassium stress [22,62,63], and the roles of these genes in the regulation of cold resistance should be analyzed in further studies.

## 4. Materials and Methods

### 4.1. Plant Materials and Sub-Optimal Temperature Treatment 

The cold-tolerant tomato cultivar Dongnong 722 (T722) and the cold-sensitive tomato cultivar Dongnong 708 (S708) used in this study were provided by the Tomato Breeding Center of Northeast Agricultural University in Harbin China. Pre-germinated seeds were sown into small pots (10 × 10 cm, one plant per pot) containing a mixture of vermiculite and perlite (volume: volume = 2:1). To avoid the influence of fertilization, each pot was irrigated once in 3 days with 100 mL of half-strength Hoagland nutrient solution that contained 2.0 mM Ca(NO_3_)_2_, 2.0 mM KNO_3_, 0.5 mM MgSO_4_, 0.5 mM NH_4_H_2_PO_4_, 0.5 mM (NH_4_)_2_HPO_4_, 0.5 mM NaCl, 20.6 μM FeNaEDTA, 6.25 μM H_3_BO_3_, 0.5 μM MnSO_4_, 0.195 μM CuSO_4_, 0.5 μM ZnCl_2_, and 0.25 μM NaMoO_4_. The pH of the nutrient solution was adjusted to 6.0 with 0.1 mM KOH. The seedlings were grown in a chamber with day/night temperatures of 25/18 °C, 65% relative humidity, and a 16 h photoperiod (irradiation intensity of 480 μmol m^−2^ s^−1^).

Once the seedlings reached the four-leaf stage, uniform-sized plants were selected for sub-optimal temperature treatment and transferred to a chamber with day/night temperatures of 15/10 °C (T) (the other parameters were not changed compared with these of the CK treatment). Two temperature treatments were used in this experiment: S708 and T722 tomato seedlings grown in the normal temperature 25/18 °C (CK) chamber were used as the control groups, and S708 and T722 plants grown in the sub-optimal temperature chamber comprised the experimental treatment groups. 

This study was performed at the Laboratory of Vegetables Physiological Economy (Harbin, China). The dry weight, K^+^ content and amount, root morphology, MDA content and POD activity of the plants were measured at 5 and 10 days after treatment (DAT). At five hours and five days after treatment, the net flux of K^+^ was measured. There were 60 pots for each treatment with a randomized arrangement resulting in a total of 120 pots (2 treatments × 2 cultivars × 3 replicates × 2 sampling times × 5 pots). The experiment was replicated three times. Since there were obvious differences in plant growth rate, root surface area and total root length of the two tomato cultivars to sub-optimal temperature at 5 DAT, so we selected the 5 DAT point as the optimum one for RNAseq analysis. At five DAT, the roots of five tomato seedlings in each treatment per replicate were harvested and pooled as one sample, with three samples per treatment (n = 3). The samples were cleaned with autoclaved deionized water, instantly frozen in liquid nitrogen and subsequently stored at −80 °C for RNA-seq library preparation [64].

### 4.2. Measurements of Plant Height, Dry Weight and Root Morphology 

Plants were harvested 1 day prior to sub-optimal temperature treatment and 5 and 10 DAT. The plant height was measured using a ruler, and the dry weight values of the leaves, stems, and roots of tomato seedlings were obtained after the samples were dried in an oven at 70 °C to a constant weight. The plant growth rate (PGR) and increase in plant height (IPH) were calculated as previously described by Beadle (1993) [65], with some modification:

PGR (g day^−1^) = (W2 − W1)/(T2 − T1)

IPH (cm day^−1^) = (H2 − H1)/(T2 − T1)

where W is the total dry weight, T is the time, H is the plant height, the subscripts 1 and 2 indicate the start and the end of the period for which the IPH and PGR were calculated.

### 4.3. Measurements of MDA Content and POD Activity

Membrane lipid peroxidation in tomato seedling roots was determined by measuring the malondialdehyde (MDA) content using the method of Dhindsa et al. (1981) [66]. Peroxidase (POD) activity was assayed according to the method of Khanna-Chopra and Chauhan (2015) [67]. 

### 4.4. Measurements of K^+^ Content, K^+^ Amount, K^+^ Net Uptake Rate and K^+^ Transportation Ratio

The dry samples were ground into powder, and digested with sulfuric acid ashed in a muffle furnace to measure the K^+^ contents of tomato seedlings, which were measured by flame spectrometry using a PerkinElmer atomic absorption spectrometer (Analyst 800). The K^+^ amount (expressed in units of mg K^+^ plant^−1^ DW) was calculated according to the method of Chachar et al. (2015) [68].

The K^+^ net uptake rate of the plant was calculated according to the following equation [69]: K^+^ net uptake rate = (C_2_ − C_1_) / [(t_2_ − t_1_) × (R_2_ + R_1_)/2], where C is the total K amount, R is the root dry weight and t is the time; the subscripts 1 and 2 indicate the start and the end of the period for which the uptake rate was calculated: t_2_−t_1_ = 5 d, and (R_2_+R_1_)/2 = the mean root dry weight.

The K^+^ transportation ratio was calculated according to the following equation [70]: K^+^ transportation ratio = 100 × (S_2_ − S_1_) / (C_2_ − C_1_), where S is the total K amount in the shoots, C is the total K^+^ uptake in the whole plants, and the subscripts 1 and 2 indicate the start and the end of the period for which the uptake rate was calculated.

### 4.5. Measurement of Net K^+^ Flux 

The net flux of K^+^ was measured at the YoungerUSA Xuyue (Beijing) BioFunction Institute using Non-invasive Micro-test Technology and iFluxes/imFluxes 1.0 Software (NMT100 Series). Tomato seedlings with four leaves were cultured in half-strength Hoagland nutrient solution. After being treated with normal and sub-optimal temperatures for 5 hours and 5 days, the tomato seedling roots were rinsed with distilled water, transferred to the measuring solution, and allowed to equilibrate for 30 min. The K^+^ measuring solution consisted of 0.1 mM KCl, 0.1 mM CaCl_2_, 0.1 mM MgCl_2_, 0.5 mM NaCl. 0.3 mM MES, and 0.2 mM Na_2_SO_4_ (pH 6.0, maintained using Tris or HCl). After primary scans along the roots, a site in the root that was 400 μm from the root tip was selected for measurement of net flux of K^+^. The glass micropipettes were prepared as previously described [71]. The data were collected every 6 s during the 10 min measurement period. Each point represents the mean of three individual plants. Positive values in the figures represent K^+^ efflux and negative values represent K^+^ influx.

### 4.6. RNA Preparation 

In the presence of liquid nitrogen, 100 mg of fresh tomato roots was ground into paste, and the total RNA from tomato roots was extracted using the TRIzol-based method described by Pattemore (2014) [72]. The RNA was quantified using an Agilent 2100 Bioanalyzer (Agilent Technologies, Santa Clara, CA, USA), and the RNA integrity and quality were evaluated using a NanoDrop spectrophotometer (Thermo Scientific, Wilmington, DE, USA).

### 4.7. Illumina Sequencing

Poly(A) mRNA was enriched using beads with Oligo (dT), and the mRNA-enriched beads were sheared into short fragments in fragmentation buffer. Using these short mRNA fragments as templates, cDNA was then synthesized. Suitable fragments were used for PCR amplification. Twelve tomato root cDNA libraries were constructed for paired-end transcriptome sequencing using the Illumina HiSeqTM 2000 system by Shanghai Majorbio Biopharm Technology Co., Ltd. (Shanghai, China). All raw data were deposited in NCBI Sequence Read Archive (SAR, http://www.ncbi.nlm.nih.gov/Traces/sra) with accession number SRP156519.

#### 4.7.1. Transcriptome Assembly and Annotation

The raw reads generated by Illumina HiSeqTM2000 were cleaned by removing adapter sequences, low-quality reads (Q < 25), reads containing more than 10% ‘N’, and fragments less than 20 bp long using SeqPrep (http://gihub.com/jstjohn/) and Sickle (http://github.com/najoshi/). 

#### 4.7.2. Analysis of Differential Gene Expression 

The clean reads were mapped to the *S. lycopersicum* reference genome sequence (http://plants.ensembl.org/Solanum_lycopersicum/Info/Index). Gene expression levels in terms of transcript abundances were quantified by RSEM (http://deweylab.biostat.wisc.edu/rsem/) and fragments per kilobase per million mapped (FPKM) [73]. The differentially expressed genes (DEGs) between the different treatments were analyzed using edgeR (http://www.bioconductor.org/packages/2.12/bioc/html/edgeR.html). Genes that exhibited a 2-fold or greater change between the two treatments and a false discovery rate (FDR) of 5% or less were defined as differentially expressed. The enrichment of DEGs was analyzed using GOATOOLS (https://github.com/tanghaibao/goatools) [74], and the GO terms with an adjusted *p*-value < 0.05 were considered significantly enriched in the DEGs. KOBAS (http://kobas.cbi.pku.edu.cn/home.do) was used for KEGG metabolic pathway analysis, and the metabolic pathways with a *p*-value < 0.05 were defined as being significantly enriched [75].

### 4.8. Verification of RNA-Seq Results by Quantitative Real-Time PCR (qRT-PCR)

Sixteen genes were selected for verification of the RNA-seq results by qRT-PCR. The gene-specific primer pairs for the *SIHAK5* reference were described by Nieves-Cordones et al. (2008) [76], and the other gene-specific primers were designed using Primer 5.0 software and synthesized by Sangon Biotech Company (Shanghai, China). These genes and their specific primers used in the real-time PCR analysis are listed in the Supplementary Materials (Table S7). Real-time quantitative RT-PCR (qRT-PCR) analyses were performed with independent samples from the S708 and T722 cultivars grown at 25/18 °C and 15/10 °C with the same conditions as those used for RNA-Seq analysis. All real-time PCR reactions were performed using the real-time PCR system qTOWER (Analytik jena, Jena, Germany). The reaction mixture, consisted of a final volume of 20 μL, which contained 10 μL of 2×SYBR Premix (BioTeke Corporation, Beijing, China, 0.3 μL of each primer, 8.8 μL of sterile deionized water, and 0.6 μL of the diluted cDNA template. The thermal cycling program comprised an initial step at 95 °C for 30 s, followed by 40 cycles at 95 °C for 20 s, 58 °C for 30 s, and 72 °C for 15 s. Each sample was analyzed with three biological replicates. The tomato *Actin* gene was used as a reference gene and the gene expression was calculated by the 2^−△△Ct^ method [77].

### 4.9. Statistical Analysis 

Mean comparison among different treatments was performed based on the Tukey’s honestly significant difference (HSD) test at the 0.05 probability level. IPH, PGR, root surface area, total root length, MDA content, POD activity, K^+^ content in roots, stems and leaves, K^+^ amount in whole plants, K^+^ net uptake rate and K^+^ transportation ratio were measured, and these data were analyzed with two-way ANOVA with cultivar, temperature and their interaction as fixed factors. These statistical analyses were conducted with SAS 9.1 software (SAS Institute Inc., Cary, NC, USA). 

## 5. Conclusions

Overall, we found some different responses to sub-optimal temperature by two tomato cultivars. Compared to cold-sensitive tomato cultivar S708, cold-tolerant cultivar T722 exhibited lower reduction in plant growth rate, the whole plant K^+^ amount and K^+^ net uptake, and T722 also had higher POD activity and lower K^+^ efflux rate under sub-optimal temperature conditions. The RNA-seq analysis showed that the overall number of DEGs at 5 DAT in T722 were higher than that in S708. GO and KEGG enrichment analysis showed that genes involved in ROS homeostasis and hormone metabolic were differentially expressed between S708 and T722 under sub-optimal temperature conditions. Up-regulation of these genes in T722 during sub-optimal temperature stress might play important roles in regulating plant root growth and cold tolerance. Moreover, we also found that sub-optimal temperature inhibited the expression of gene coding for K^+^ channel AKT1, which is associated with K^+^ uptake only in S708, might be a reason for less K^+^ amount than T722. The results revealed the cold response genes in tomato roots, and provided a foundation for further investigating the mechanism of K^+^ uptake in tomato under sub-optimal temperatures.

## Figures and Tables

**Table 1:** *F*-value from two-way ANOVA analysis of effects of cultivar and temperature treatment on IPH, PGR, root surface area, total root length, MDA content and POD activity.

DAT	Variables	IPH	PGR	Root Surface Area	Total Root Length	MDA Content	POD Activity
5	Cultivar (C)	23.74 **	26.60 **	2.81 ns	77.94 ***	13.64 *	107.19 ***
	Temperature (T)	113.77 ***	53.86 ***	40.71 ***	72.83 ***	62.28 **	740.64 ***
	C × T	3.40 ns	12.75 *	4.88 ns	13.48 **	23.23 **	70.22 ***
10	Cultivar (C)	30.77 **	11.34 *	30.09 **	4.30 ns	4.25 ns	52.14 ***
	Temperature (T)	296.34 ***	181.36 ***	93.44 ***	114.79 ***	34.88 **	390.29 ***
	C × T	16.13 **	16.93 **	1.91 ns	3.79 ns	3.81 ns	42.14 ***

ns not significant. * *p* ≤ 0.05, ** *p* ≤ 0.01, *** *p* ≤ 0.001.

**Table 2:** K^+^ content in roots, stems and leaves and K^+^ amount in whole plants of the S708 and the T722 cultivar under normal (CK, 25/18 °C) and sub-optimal temperature (T, 15/10°C) conditions at 5 and 10 DAT. Data are the means with standard deviation (n = 3 representing 3 biological replicates). Different letters on the same column indicate significant differences attested by the Tukey’s HSD test (*p* < 0.05). *F*-value from two-way ANOVA analysis of effects of cultivar and temperature treatment on K^+^ content in roots, stems and leaves and K^+^ amount in whole plants.

DAT	Treatments	K^+^ Content (mg g^−1^)			K^+^ Amount (mg Plant^−1^)
		Root	Stem	Leaf	
5	S708 CK	23.76 ± 0.60 b	46.69 ± 0.55 b	25.11 ± 1.55 ab	15.45 ± 0.50 c
	S708 T	28.17 ± 1.31 a	42.62 ± 1.30 c	22.68 ± 0.52 b	8.48 ± 0.70 d
	T722 CK	23.69 ± 1.60 b	52.38 ± 0.96 a	26.55 ± 1.73 a	26.01 ± 1.01 a
	T722 T	27.40 ± 0.98 a	49.24 ± 0.92 b	26.18 ± 1.15 ab	21.52 ± 0.87 b
Analysis of variance					
Cultivar (C)		ns	***	*	***
Temperature (T)		**	***	ns	***
C × T		ns	ns	ns	ns
10	S708 CK	26.70 ± 2.14	50.76 ± 2.38 a	27.46 ± 0.78 a	27.85 ± 0.98 c
	S708 T	29.07 ± 1.44	43.96 ± 1.51 b	22.90 ± 1.11b	17.55 ± 0.54 d
	T722 CK	24.68 ± 1.18	51.61 ± 0.74 a	27.66 ± 0.78 a	41.44 ± 0.39 a
	T722 T	26.17 ± 0.79	49.62 ± 0.74 a	26.48 ± 1.22 a	33.53 ± 1.23 b
Analysis of variance					
Cultivar (C)		ns	*	*	***
Temperature (T)		ns	**	**	***
C × T		ns	ns	*	ns

ns not significant. * *p* ≤ 0.05, ** *p* ≤ 0.01, *** *p* ≤ 0.001.

**Table 3:** Significantly enriched KEGG pathway of differentially expressed genes (DEGs) in response to sub-optimal temperature (T, 15/10 °C).

	Pathway	Number of Up-Regulated Genes	Number of Down-Regulated Genes	Pathway ID
S708 CK-S708 T	Plant hormone signal transduction	17	7	ko04075
	Phenylpropanoid biosynthesis	13	15	ko00940
	Sulfur metabolism	8	0	ko00920
	Cytochrome P450	8	5	ko00982
T722 CK-T722 T	Plant hormone signal transduction	21	17	ko04075
	Phenylpropanoid biosynthesis	24	18	ko00040
	Sulfur metabolism	8	2	ko00920
	Cytochrome P450	8	8	ko00982

**Table 4:** List of selected genes induced by sub-optimal temperature (T, 15/10 °C) in the significantly enriched KEGG pathway “plant hormone signal transduction” only in T722. S708 and T722 tomato cultivars grown under normal (CK, 25/18 °C) and sub-optimal temperature conditions. Values are the log_2_ratio (T/CK) for genes more than 1 or less than −1 with FDR < 0.05 as the threshold to judge the significance of gene expression difference between the two groups.

Gene ID	S708	T722	Annotation
	log_2_(T/CK)	log_2_(T/CK)	
**Auxin**			
Solyc09g065850.2	0.72	1.40	IAA3 protein, AUX/IAA (*S*. *lycopersicum*)
Solyc04g081270.1	0.03	1.28	Indole-3-acetic acid-induced protein, ARG7 SAUR (*S. lycopersicum*)
**Gibberellin**			
Solyc09g074270.2	0.93	1.05	Gibberellin receptor, GID1 (*S*. *lycopersicum*)
**Abscisic acid**			
Solyc10g050210.1	0.56	1.44	Abscisic acid insensitive, ABF (*S*. *lycopersicum*)
**Ethylene**			
Solyc12g009560.1	0.87	1.18	EIN3-binding F-box protein, EBF1 (*A*. *thaliana*)
**Brassinosteroid**			
Solyc04g078470.2	0.56	1.16	CycD3;2 protein (*A*. *thaliana*)
**Jasmonic acid**			
Solyc08g008600.2	0.46	2.19	Transcription factor, MYC2 (*S*. *lycopersicum*)
**Salicylic acid**			
Solyc01g106640.2	0.06	1.50	Pathogenesis-related protein, PR1 (*S*. *lycopersicum*)
Solyc10g085960.1	0.64	1.22	Pathogenesis-related protein, PR1 (*S*. *tuberosum*)
Solyc09g007020.1	−0.31	1.24	Pathogenesis-related protein, PR1 (*S*. *lycopersicum*)

**Table 5:** List of selected genes induced by sub-optimal temperature in the significantly enriched KEGG pathway “phenylpropanoid biosynthesis” only in T722. S708 and T722 tomato cultivars grown under normal (CK, 25/18 °C) and sub-optimal temperature conditions. Values are the log_2_ratio (T/CK) for genes more than 1 or less than −1 with FDR < 0.05 as the threshold to judge the significance of gene expression difference between the two groups.

Gene ID	S708	T722	Annotation
	log_2_(T/CK)	log_2_(T/CK)	
Solyc11g018800.1	0.86	1.17	Lignin-forming anionic peroxidase (*S*. *lycopersicum*)
Solyc10g078890.1	0.07	1.30	Peroxidase (*S*. *lycopersicum*)
Solyc05g050880.2	0.02	1.86	Cationic peroxidase (*S. lycopersicum*)
Solyc12g096530.1	0.92	1.29	Peroxidase (*A. thaliana*)
Solyc02g085930.2	0.99	1.36	Peroxidase (*A*. *thaliana*)
Solyc02g077300.1	0.61	1.02	Peroxidase (*A*. *thaliana*)
Solyc03g120800.2	0.61	1.55	Peroxidase (*A*. *thaliana*)
Solyc10g076210.1	0.35	1.14	Peroxidase (*S*. *lycopersicum*)
Solyc01g058520.2	0.57	1.16	Peroxidase (*S*. *lycopersicum*)
Solyc01g101050.2	0.57	1.55	Peroxidase (*S*. *lycopersicum*)
Solyc05g050870.2	−0.09	1.48	Cationic peroxidase (*S*. *tuberosum*)
Solyc05g050890.1	0.38	1.92	Cationic peroxidase (*S*. *lycopersicum*)

**Table 6:** List of genes related to K^+^ uptake and translocation in the S708 and the T722 cultivar under sub-optimal temperature conditions (T, 15/10 °C). S708 and T722 tomato cultivars grown under normal (CK, 25/18 °C) and sub-optimal temperature conditions. Values are the log_2_ratio (T/CK) for genes more than 1 or less than -1 with FDR < 0.05 as the threshold to judge the significance of gene expression difference between the two groups.

Gene ID	S708	T722	Annotation
	log_2_(T/CK)	log_2_(T/CK)	
Solyc09g042660.2	0.99	1.03	CBL-interacting protein kinase 18 OsCIPK18 (*O*. *sativa*)
Solyc03g006110.2	−0.02	2.04	CBL-interacting protein kinase 5 OsCIPK05 (*O*. *sativa*)
Solyc03g083320.2	0.94	1.24	Calcineurin B-like protein 7 OsCBL7 (*O*. *sativa*)
Solyc09g005220.1	−1.56	−0.40	Potassium channel AKT1 (*A. thaliana*)
Solyc12g006850.1	−0.94	−0.36	Potassium channel LKT1 (*S. lycopersicum*)
Solyc12g005670.1	−1.07	−1.07	Potassium transporter 5 SIHAK5 (*S. lycopersicum*)
Solyc06g051830.1	−3.39	−1.45	Potassiumtransporter HAK26 (*O*. *sativa*)
Solyc08g007060.2	−1.26	−1.55	Nitrate transporter 1.5 AtNRT1.5 (*A. thaliana*)

## Supplementary Materials

The following are available online at https://www.mdpi.com/2223-7747/9/1/65/s1, Table S1: Major characteristic of twelve libraries. Table S2: The DEGs in the S708 cultivar. Table S3: The DEGs in the T722 cultivar. Table S4: Significantly enriched GO biological process terms of DEGs in both tomato cultivars under sub-optimal temperature stress. Table S5: KEGG pathway enrichment analysis of DEGs. Table S6: List of aquaporins and cold tolerance related genes in the S708 and T722 cultivar under sub-optimal temperature conditions. Table S7: Primers used in qPCR experiments.
