# Supplementary material for: Gene Expression and K+ Uptake of Two Tomato Cultivars in Response to Sub-Optimal Temperature
Source: Plants (Basel). 2020 Jan 3;9(1):65. doi: 10.3390/plants9010065 (PMC7020494; doi:10.3390/plants9010065)
Supplement: Supplementary file 1 [file plants-09-00065-s001.zip › Supplementary Material/Table S1.docx]

**Table S1.** Major characteristic of twelve libraries.

|  | **Raw reads** | **Clean reads** | **Mapped reads** | **Mapping rate** |
| --- | --- | --- | --- | --- |
| S708 CK1 | 56717588 | 55453862 | 52782649 | 95.18% |
| S708 CK2 | 52956726 | 52956726 | 49309451 | 95.10% |
| S708 CK3 | 47604846 | 47604846 | 44601839 | 95.35% |
| S708 T1 | 55512630 | 54506628 | 52213252 | 95.79% |
| S708 T2 | 64664382 | 63369116 | 60557896 | 95.56% |
| S708 T3 | 53279216 | 52104160 | 49881316 | 95.73% |
| T722 CK1 | 54059500 | 52951304 | 50601202 | 95.56% |
| T722 CK2 | 57495436 | 56471824 | 54119014 | 95.83% |
| T722 CK3 | 69453098 | 68059980 | 65279322 | 95.91% |
| T722 T1 | 53486516 | 52530884 | 50213061 | 95.59% |
| T722 T2 | 45989114 | 45065298 | 43138925 | 95.73% |
| T722 T3 | 53666134 | 52781700 | 50597225 | 95.86% |
