# Supplementary material for: Gene Expression and K+ Uptake of Two Tomato Cultivars in Response to Sub-Optimal Temperature
Source: Plants (Basel). 2020 Jan 3;9(1):65. doi: 10.3390/plants9010065 (PMC7020494; doi:10.3390/plants9010065)
Supplement: Supplementary file 1 [file plants-09-00065-s001.zip › Supplementary Material/Table S6.docx]

| GenBank accession no. | Sequence of primers（5’-3’） | Annotation |
| --- | --- | --- |
| Solyc04g011500.2 | GAAATAGCATAAGATGGCAGACG  ATACCCACCATCACACCAGTAT | Actin |
| Solyc09g005220.1 | GCGTGTCACAGTCCTACAA  CTCTGTAGCAACCGCGTGTA | Potassium channel AKT1 (*A. thaliana*) |
| Solyc12g005670.1 | CCGTGTTACGCGCCTTTAA  TCCTTTTTTACCGTTTCTTTTGAAG | Potassium transporter 5 LeHAK5  (*S. lycopersicum*) |
| Solyc09g042660.2 | TGTCAAAGGAGGCGAGTTGT  TGCAAGGGCACTCAATCCAA | CBL-interacting protein kinase 18 OsCIPK18 (*O*. *sativa*) |
| Solyc03g006110.2 | CGGGACGGTGAAAATGCAAG  TCGTGAGCTGGTAGGTCTCA | CBL-interacting protein kinase 5  OsCIPK05 (*O*. *sativa*) |
| Solyc03g083320.2 | GCTGCTGAGACCGCTTTTAC  AATCACTCCGTTGCGCTTGA | Calcineurin B-like protein 7  (*O*. *sativa*) |
| Solyc12g006850.1 | TGGGAGGCTATCATGGGGAA  TCGTGCTCATTGGTCTGCTT | Potassium channel LKT1  (*S. lycopersicum*) |
| Solyc06g051830.1 | CTCACTCCTGCTACTTGCGT  ATAAGCCGTTTCTCCGGCAT | Potassium transporter HAK26  (*O*. *sativa*) |
| Solyc08g007060.2 | TGGCGTCTCTGCCCTATTTC  AGGCCATCTGCGTGAAAACT | Nitrate transporter 1.5 AtNPF7.3  (*A. thaliana*) |
| Solyc10g083880.1 | GCGATAGCCCATGCTTTTGG  GCAACAACAGAGCCAAGCAA | Aquaporin TIP1-1 (*A. thaliana*) |
| Solyc10g054800.1 | ACTCTTGGCGGTTGTTCCAG  ATAATGCTTGGGCCGTCTCT | Aquaporin PIP1-6 (*S. lycopersicum*) |
| Solyc10g011920.1 | TCCGAAACAGTCACATCGCA  AGCATCTTCCGCGCTATCAA | Phenylalanine ammonia-lyase 1 PAL  (*S. lycopersicum*) |
| Solyc01g067850.2 | GTCAGGGGTTGTGATGGTTCA  GTATTGCAGCCTTCTCTGCAC | Peroxidase 19-like (*S. lycopersicum*) |
| Solyc01g006300.2 | AGTACCGATGTTCGTGCTGG  GACACACGTTTTCCAACGCA | Peroxidase (*S. lycopersicum*) |
| Solyc09g089930.1 | TCCGAAACAGTCACATCGCA  AGCATCTTCCGCGCTATCAA | Ethylene-responsive transcription factor 1B TSRF1 (*A. thaliana*) |
| Solyc03g005520.1 | CCTTCTCGCACAAGCTGACT  GAATCTCTAATCTCCGCCGCA | Ethylene-responsive transcription factor 1B AtERF1B (*A. thaliana*) |
| Solyc06g008440.1 | TGCCCTAAGCTTCAGTCAGT  CATAGATCGCTATCCCCCGC | Ethylene-insensitive protein 1 TIR1  *(A. thaliana*) |

**Table S6.** Primers used in qPCR experiments
