# Supplementary material for: Gene Expression and K+ Uptake of Two Tomato Cultivars in Response to Sub-Optimal Temperature
Source: Plants (Basel). 2020 Jan 3;9(1):65. doi: 10.3390/plants9010065 (PMC7020494; doi:10.3390/plants9010065)
Supplement: Supplementary file 1 [file plants-09-00065-s001.zip › Supplementary Material/Table S5.doc]

| Gene ID | S708 | T722 | Annotation |
| --- | --- | --- | --- |
|  | log_2_(T/CK) | log_2_(T/CK) |  |
| Solyc10g083880.1 | -1.81 | -2.20 | Aquaporin TIP1-1-like (*S. lycopersicum*) |
| Solyc10g054800.1 | -2.43 | -2.20 | Aquaporin PIP1-6-like (*S. lycopersicum*) |
| Solyc02g089630.2 | -1.65 | -2.99 | Proline dehydrogenase 2 (*S. lycopersicum*) |
| Solyc02g089620.2 | -0.91 | -1.66 | Proline dehydrogenase 1 (*S. lycopersicum*) |
| Solyc12g005360.1 | 0.11 | 1.1 | Mitogen-activated protein kinase ANP1-like (*S. lycopersicum*) |
| Solyc02g090980.1 | 0.94 | 1.47 | Mitogen-activated protein kinase kinase kinase A (*S. lycopersicum*) |
| Solyc05g008020.2 | 0.03 | 1.27 | Mitogen-activated protein kinase 15 (*S. lycopersicum*) |

**Table S5.** List of aquaporin genes and cold tolerance related genes in the S708 and T722 cultivar under sub-optimal temperature conditions. S708 and T722 tomato cultivars grown at normal (CK, 25/18°C) and sub-optimal temperature (T, 15/10°C) conditions. Values are the log_2_ratio (T/CK) for genes more than 1 or less than -1 with FDR<0.05 as the threshold to judge the significance of gene expression difference between the two groups.

**Table S4.** List of cold response genes in S708 and T722c under sub-optimal temperature conditions. 708 and 722 tomato cultivars grown at normal (CK, 25/18°C) and sub-optimal temperature (T, 15/10°C) conditions. Values are the log_2_ratio (T/CK) for genes more than 1 or less than -1 with FDR<0.05 as the threshold to judge the significance of gene expression difference between the two groups.

| Gene ID | 708 | 722 | Annotation |
| --- | --- | --- | --- |
|  | log_2_(T/CK) | log_2_(T/CK) |  |
| Solyc10g083880.1 | -1.81 | -2.20 | Aquaporin TIP1-1-like (*S. lycopersicum*) |
| Solyc10g054800.1 | -2.43 | -2.20 | Aquaporin PIP1-6-like (*S. lycopersicum*) |
| Solyc02g089630.2 | -1.65 | -2.99 | Proline dehydrogenase 2 (*S. lycopersicum*) |
| Solyc02g089620.2 | -0.91 | -1.66 | Proline dehydrogenase 1 (*S. lycopersicum*) |
| Solyc03g026280.2 | 0.49 | 1.02 | Dehydration-responsive element-binding protein, CBF1 (*S. lycopersicum*) |
| Solyc03g026270.1 | 1.67 | 0.95 | Dehydration-responsive element-binding protein, CBF3 (*S. lycopersicum*) |
| Solyc12g005360.1 | 0.11 | 1.1 | Mitogen-activated protein kinase ANP1-like (*S. lycopersicum*) |
| Solyc02g090980.1 | 0.94 | 1.47 | Mitogen-activated protein kinase kinase kinase A (*S. lycopersicum*) |
| Solyc05g008020.2 | 0.03 | 1.27 | Mitogen-activated protein kinase 15 (*S. lycopersicum*) |
